# Supplementary material for: Juxtaposition of Bub1 and Cdc20 on phosphorylated Mad1 during catalytic mitotic checkpoint complex assembly
Source: Nat Commun. 2022 Oct 26;13:6381. doi: 10.1038/s41467-022-34058-2 (PMC9605988; doi:10.1038/s41467-022-34058-2)
Supplement: Supplementary file 1 — Supplementary Information [file 41467_2022_34058_MOESM1_ESM.pdf]

Supp Fig 1

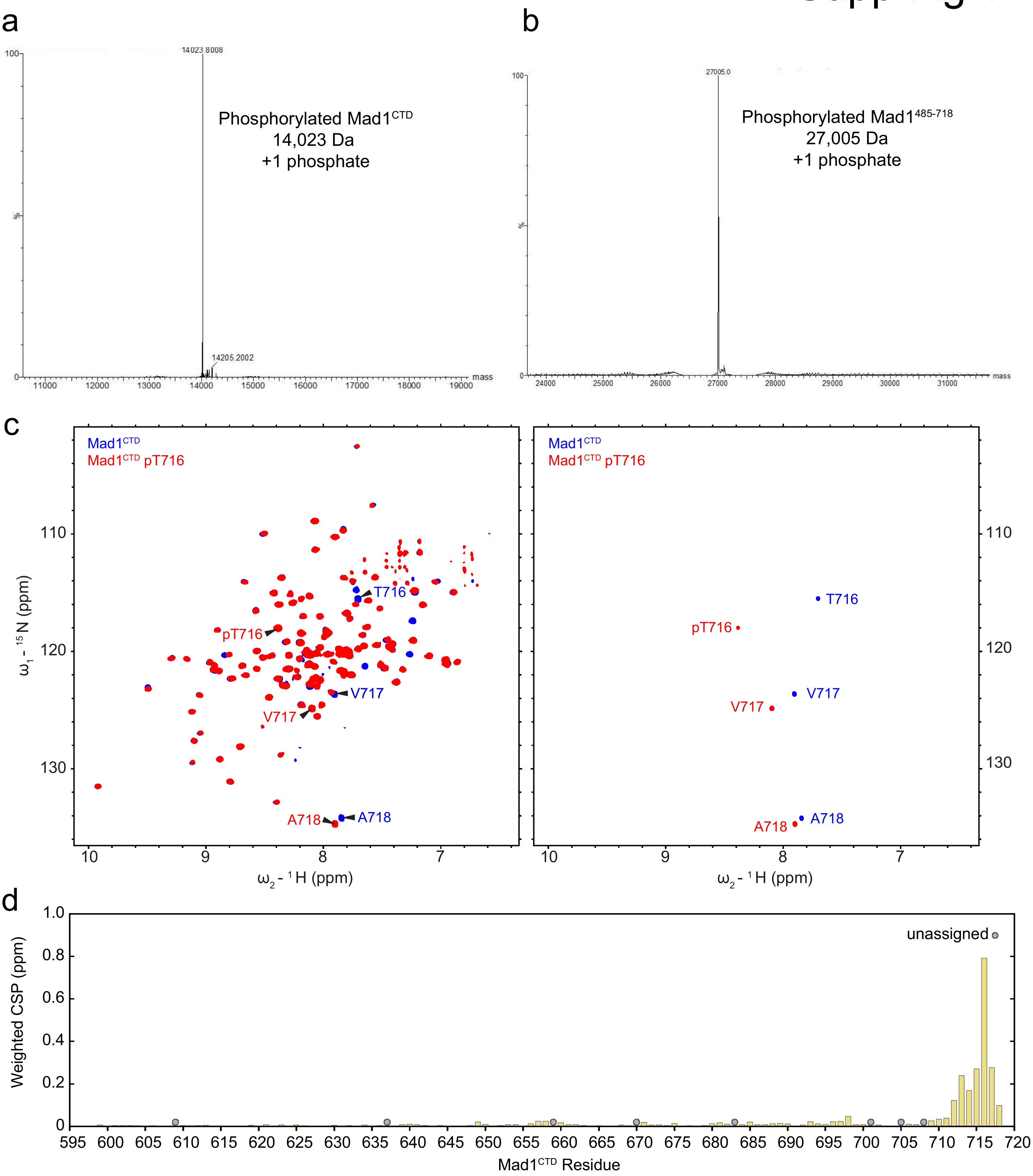
a
14023.8008
Phosphorylated Mad1CTD
14,023 Da
+1 phosphate
14205.2002
mass
b
27005.0
Phosphorylated Mad1485-718
27,005 Da
+1 phosphate
mass
c
Mad1CTD
Mad1CTD pT716
T716
pT716
V717
V717
A718
A718
ω1 - 15N (ppm)
ω2 - 1H (ppm)
d
unassigned
Weighted CSP (ppm)
Mad1CTD Residue

Supp Fig 1: Characterization of pMad1^{CTD} by intact mass spectrometry and NMR. a Mad1^{CTD} (residues 597-718) was phosphorylated by GST-Mps1 and then analysed by intact mass spectrometry. The apparent mass of the pMad1^{CTD} monomer is 14,023 Da, which is 80 Da larger than that of Mad1^{CTD} (13,943 Da), suggesting that only one residue of monomeric Mad1^{CTD} was phosphorylated by Mps1 under these conditions *in vitro.* **b** The Mad1$^{485\text{-}718}$:C-Mad2 complex was phosphorylated by GST-Mps1 *in vitro* and then analysed by intact mass spectrometry. The apparent mass of the pMad1$^{485\text{-}718}$ monomer is 27,005 Da, 80 Da larger than that of Mad1$^{485\text{-}718}$ (26,925 Da), suggesting that like Mad1^{CTD}, only one residue of monomeric Mad1$^{485\text{-}718}$ was phosphorylated by Mps1 under these conditions *in vitro.* **c** ^{1}H,^{15}N 2D HSQC showing ^{15}N-labelled Mad1^{CTD} (blue) and pMad1^{CTD} pT716 (red), shown with low (left) and high (right) contour levels. Arrows indicate the three C-terminal residues, including the phosphorylation site Thr716, identified using backbone resonances reported in our previous study[21]. As they are located in the highly flexible C-terminus, their corresponding peaks have high signal-to-noise ratios (right) and can be used to determine the extent of pThr716 phosphorylation. **d** Chemical shift perturbations observed in (c) were mapped onto the sequence of Mad1^{CTD}. Unassigned peaks are denoted as grey circles.

Supp Fig 2

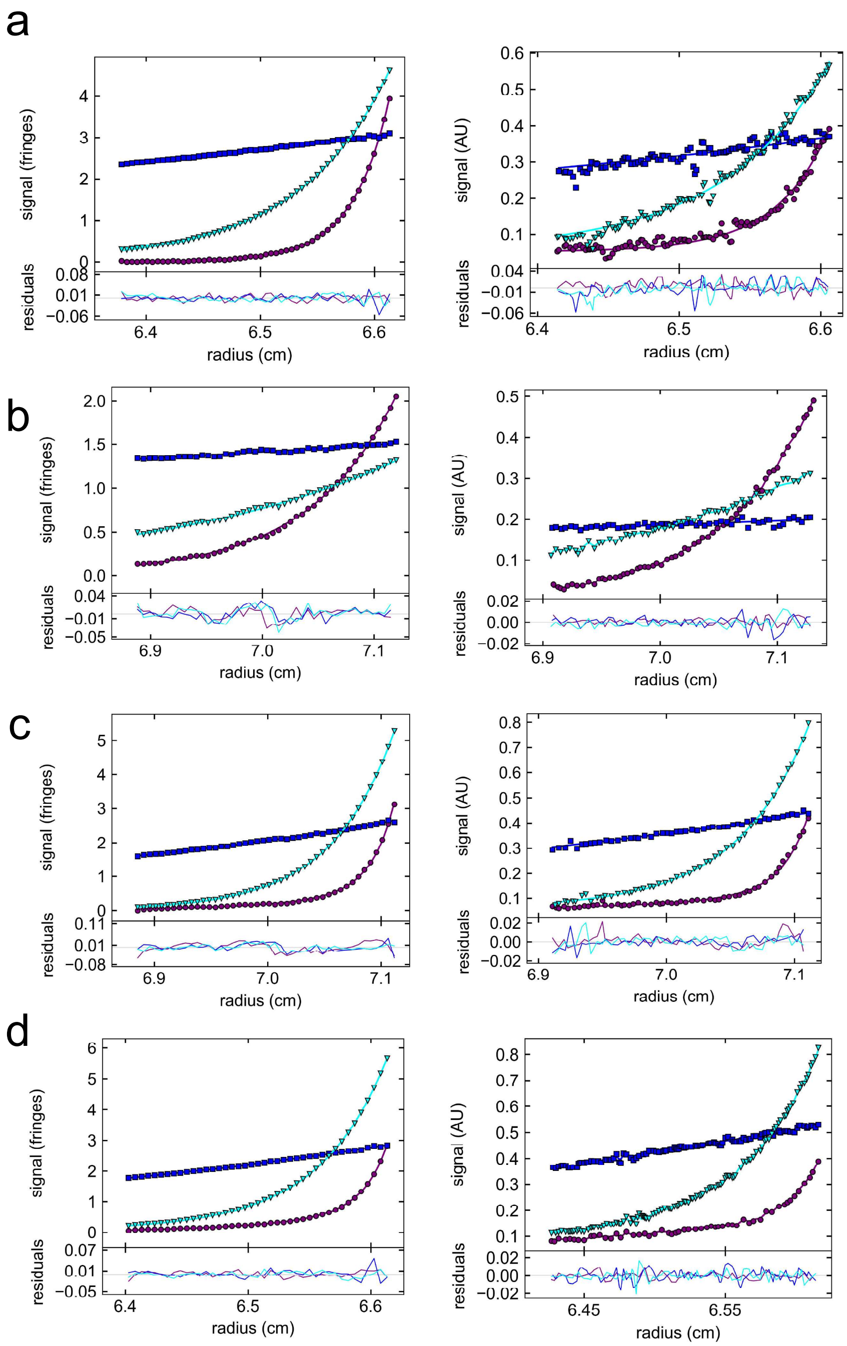

a: 20 μM Mad1^{CTD}
Mass = 26,341 Da

b: 100 μM Cdc20^{N}
Mass = 7,780 Da

c: 20 μM Mad1^{CTD}
40 μM Cdc20^{N}
Masses = 32,554 Da
and 7,786 Da*

d: 20 μM Mad1^{CTD}
80 μM Cdc20^{N}
Masses = 35,936 Da
and 7,876 Da*

Supp Fig 2: Sedimentation equilibrium (AUC-SE) of pMad1^{CTD} and Cdc20^{N}. The interference data and absorbance data are shown on the left and right panels respectively. Scans were initially fitted to single species exponentials. *When there was found to be a poor fit, the number of species was increased to two or three, and the mass of a known species (Cdc20^{N}: 7876 Da) was fixed in the fit. **a** The calculated mass for 20 µM Mad1^{CTD} pT716 was 26.3 kDa, comparable to the theoretical mass of 28 kDa for Mad1^{CTD} pT716 dimer. **b** The calculated mass for 100 µM Cdc20^{N} was 7.8 kDa, in agreement to the theoretical mass of 7.8 kDa. **c,d** The calculated mass for the major species in 20 µM Mad1^{CTD} + 40 µM Cdc20^{N} was 32.6 kDa (c) and 20 µM Mad1^{CTD} + 80µM Cdc20^{N} was 35.9 kDa (d) which are close to the expected value of a single Cdc20^{N} molecule bound to the Mad1^{CTD} dimer (35.7 kDa). The average mass for the Mad1^{CTD}:Cdc20^{N} complex across all four technical replicates was 34.7 ± 0.7 µM.

Supp Fig 3

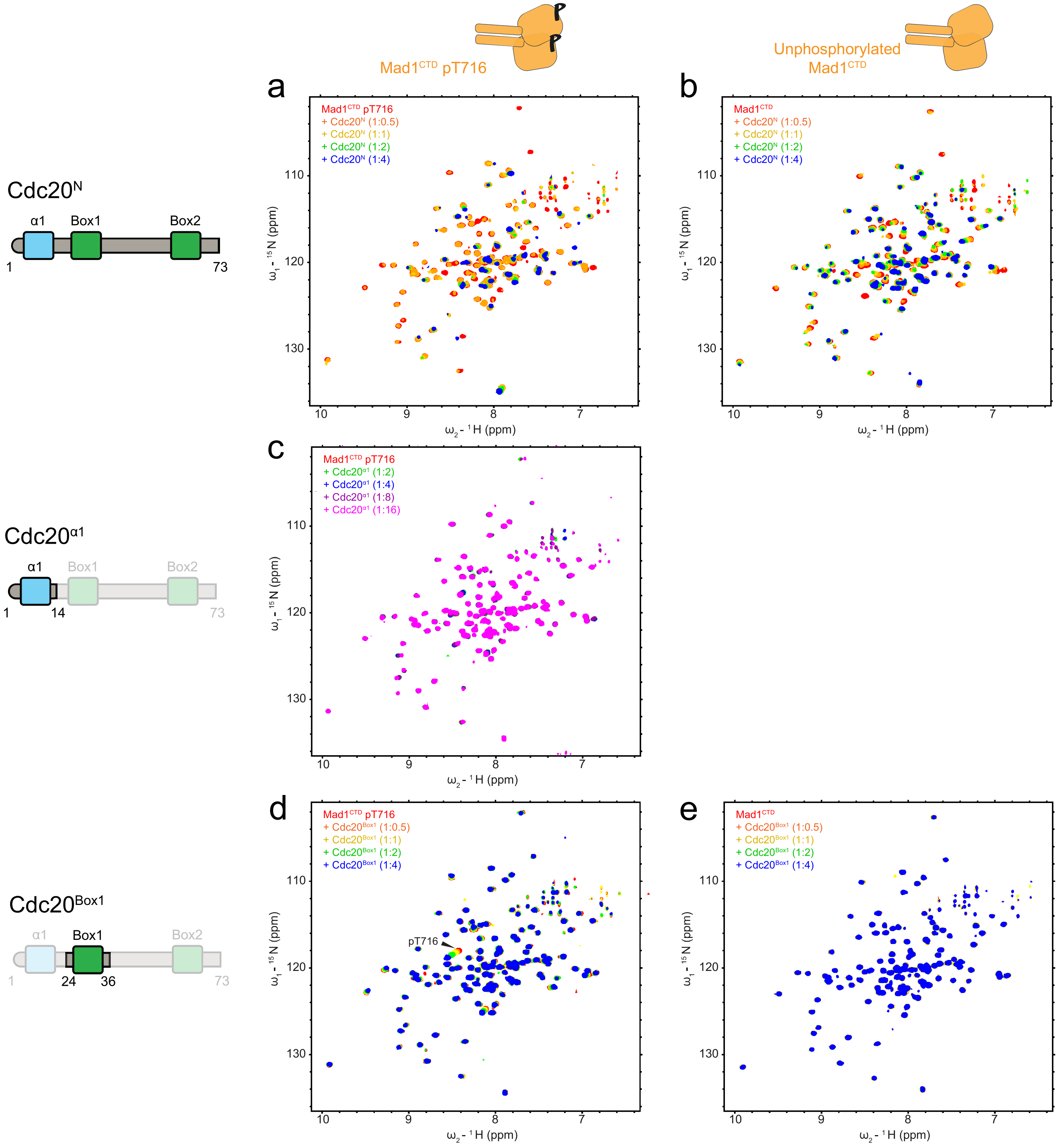

Mad1CTD pT716
Unphosphorylated Mad1CTD
a
b
c
d
e
Cdc20N
α1
Box1
Box2
1
73
Cdc20α1
14
Cdc20Box1
24
36
Mad1CTD pT716
+ Cdc20N (1:0.5)
+ Cdc20N (1:1)
+ Cdc20N (1:2)
+ Cdc20N (1:4)
Mad1CTD
+ Cdc20α1 (1:2)
+ Cdc20α1 (1:4)
+ Cdc20α1 (1:8)
+ Cdc20α1 (1:16)
+ Cdc20Box1 (1:0.5)
+ Cdc20Box1 (1:1)
+ Cdc20Box1 (1:2)
+ Cdc20Box1 (1:4)
pT716
ω1 - 15N (ppm)
ω2 - 1H (ppm)
110
120
130
10
9
8
7

Supp Fig 3: NMR spectra showing titrations of Cdc20 constructs to $Mad1^{CTD}$.

Cdc20 constructs were titrated into ^{15}N-labelled phosphorylated or unphosphorylated $Mad1^{CTD}$ and analysed in ^{1}H, ^{15}N 2D HSQC. Schematics of $Mad1^{CTD}$, $Cdc20^{N}$ and its specific domains indicate the analyte (top) and titrant (left) used in each experiment. **a,b** Titrating $Cdc20^{N}$ into $Mad1^{CTD}$ pT716 (a) or unphosphorylated $Mad1^{CTD}$ (b). **c** Titrating $Cdc20^{\alpha 1}$ into $Mad1^{CTD}$ pT716. **d,e** Titrating $Cdc20^{Box1}$ into $Mad1^{CTD}$ pT716 (d) or unphosphorylated $Mad1^{CTD}$ (e).

Supp Fig 4

a

PSIPRED α1 α2

Box1

H.sapiens	1	MAQFAFESDLHSLLQLDAPIPNAPPARWQRKAKEAAGPAP----	40
M.musculus	1	MAQFVFESDLHSLLQLDAPIPNAPVARWQRKAKEATGPAP----	40
G.gallus	1	MAHFVFEADLHGLLKLDTPIPNAPPARWQRKAKESACPGPGPSP	44
C.porcellus	1	MAQFVFESDLHSLLQLDAPIPNAPIARWQRKAKEAPGPGP----	40
X.laveis	1	MAQFVFETDINSILKLDTPITNAPLARWQRKAKEGNCSSLNTSA	44
D.melanogaster	1	MSQFNFVSDLQNALIMDGETR-GPAPRWKKKLEASLNGSVN---	40
S.pombe	11	SPTFSTPTKKRNLVFPNSPITPLHQQALLGRNGRSSKRCSPK--	52
C.elegans	2	NNKGRTPGSAGRTVRSSAQQNGLTMRKRDMTPTRNTNLLPNATF	45
S.cerevisiae	8	KGNAAISGNRSVLSIASPTKLNILSSDWSRNQGKVSKNSLKRSS	51

PSIPRED

Box2

H.sapiens	41	-----SPMRAANRSHSAGRTPGRTPGKSSSKVQTTPSKPG	73
M.musculus	41	-----SPMRAANRSHSAGRTPGRTPGKSSSKVQTTPSKPG	73
G.gallus	45	-AASMSPMKPANRSYSGSKTPSKTPGKSGSKVQSTPTKAG	83
C.porcellus	41	-----SPMRAANRSHSAGRTPGRTPGKSNSKVQTTPSKPG	68
X.laveis	45	NTSTLSPMKASNRSHSSSKTPSKTPGKSGPKMQGTPSRAG	85
D.melanogaster	41	-----TTRSVLSVSYNTSFSGVQAP---------------	60
S.pombe	53	-----SSFIRNSPKIDVVNTDWSIPLCGSPRNKSRPASRS	87
C.elegans	46	VG---DRFLGVRLDQDELDHANHLMTSKLYSNKENLNNSM	82
S.cerevisiae	52	S-----LNIRNSKRPSLQASANSIYSRPKITIGAPPLIRR	86

b

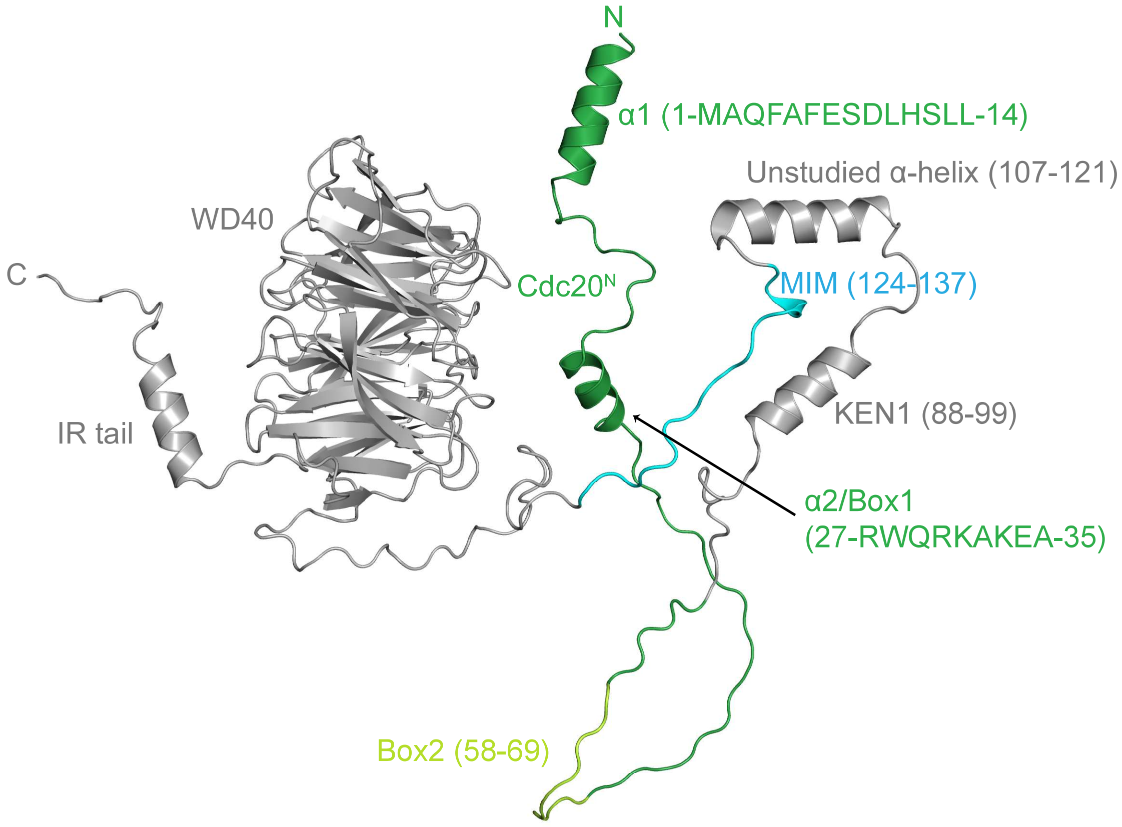

Supp Fig 4: Sequence alignment and structure prediction of Cdc20. a A multiple sequence alignment of $Cdc20^N$. The conserved Box1 and Box2 motifs are highlighted by the dashed boxes. Secondary structure prediction by PSIPRED is shown above. **b** Structure prediction of full-length Cdc20 using AlphaFold2. $Cdc20^N$ is coloured in green, Box2 which has no predicted secondary structure is coloured in light green, the MIM is coloured in cyan and the rest of Cdc20 is coloured in grey.

Supp Fig 5

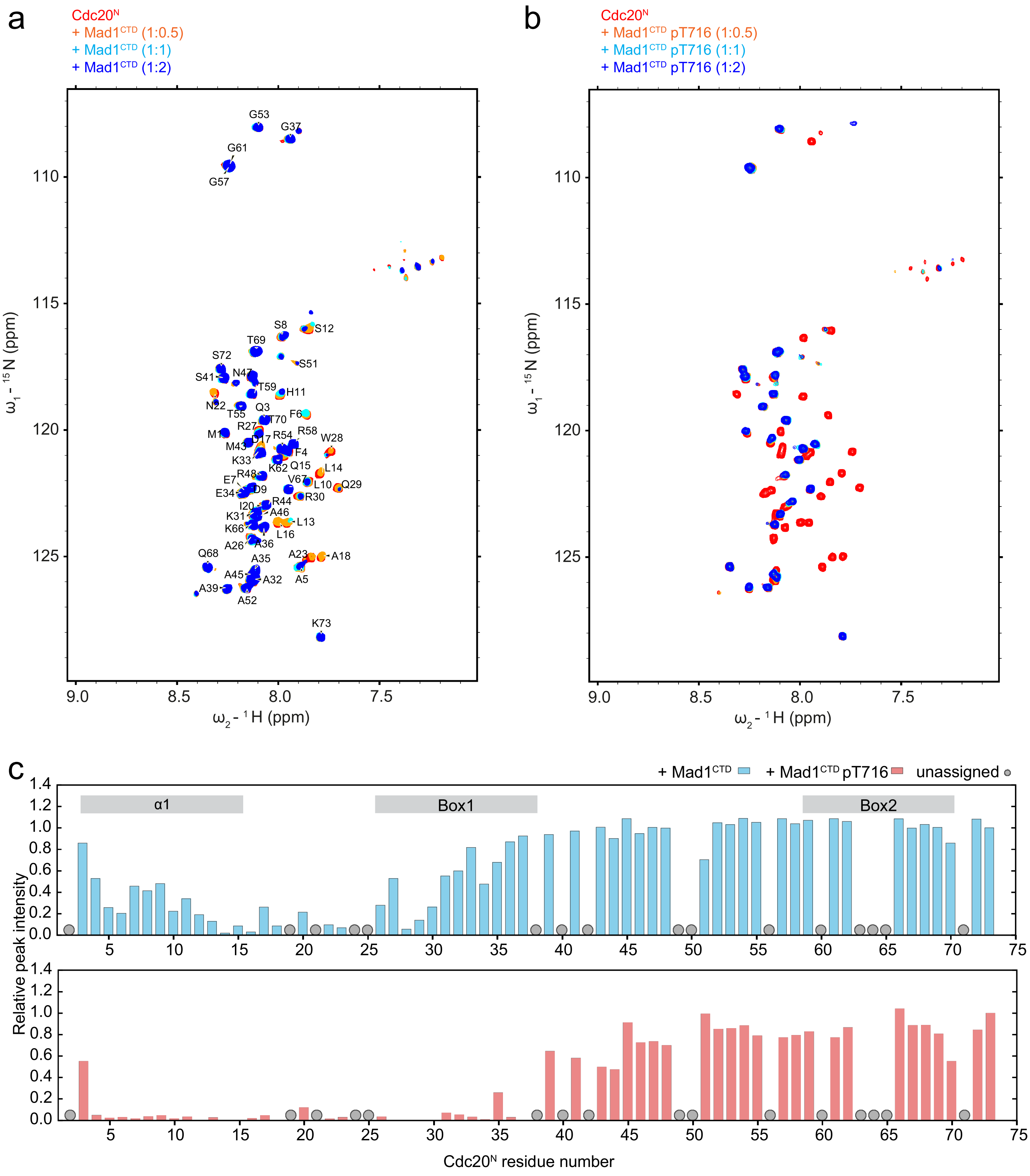

a
Cdc20ᴺ
+ Mad1ᶜᵀᴰ (1:0.5)
+ Mad1ᶜᵀᴰ (1:1)
+ Mad1ᶜᵀᴰ (1:2)
b
Cdc20ᴺ
+ Mad1ᶜᵀᴰ pT716 (1:0.5)
+ Mad1ᶜᵀᴰ pT716 (1:1)
+ Mad1ᶜᵀᴰ pT716 (1:2)
G53
G37
G61
G57
S8
S12
T69
S72
S51
N47
S41
T59
H11
N22
Q3
T55
F6
T70
R27
R58
M1
R54
W28
M43
D17
F4
K33
K62
Q15
L14
R48
V67
E7
L10
Q29
E34
D9
R30
I20
R44
K31
A46
L13
K66
L16
A26
A36
Q68
A35
A23
A18
A45
A32
A5
A39
A52
K73
110
115
120
125
9.0
8.5
8.0
7.5
ω1 - 15N (ppm)
ω2 - 1H (ppm)
c
+ Mad1ᶜᵀᴰ
+ Mad1ᶜᵀᴰ pT716
unassigned
α1
Box1
Box2
Relative peak intensity
Cdc20ᴺ residue number

Supp Fig 5: Characterization of Cdc20^{N}-Mad1^{CTD} interaction using ^{1}H-detected NMR. a ^{1}H,^{15}N 2D HSQC showing the titration of unphosphorylated Mad1^{CTD} into ^{15}N-labelled Cdc20^{N}. Backbone resonances of Cdc20^{N} are labelled with their assignment. **b** ^{1}H,^{15}N 2D HSQC showing the titration of Mad1^{CTD} pT716 into ^{15}N-labelled Cdc20^{N}. **c** Relative peak intensities from the ^{1}H,^{15}N 2D HSQC of Cdc20^{N} in the presence of phosphorylated Mad1^{CTD} (blue) or Mad1^{CTD}pT716 (red) were mapped onto the Cdc20^{N} sequence. Mad1^{CTD} dimer and Cdc20^{N} were mixed at 1:1 molar ratio. Peak intensities were normalized to the C-terminal residue Gly73. Multiple backbone resonances including those of prolines were absent in the ^{1}H,^{15}N 2D HSQC and therefore unassigned (grey circles). To achieve a more complete analysis of this interaction, ^{13}C-detected NMR were employed as shown in Fig 3a-c.

Supp Fig 6

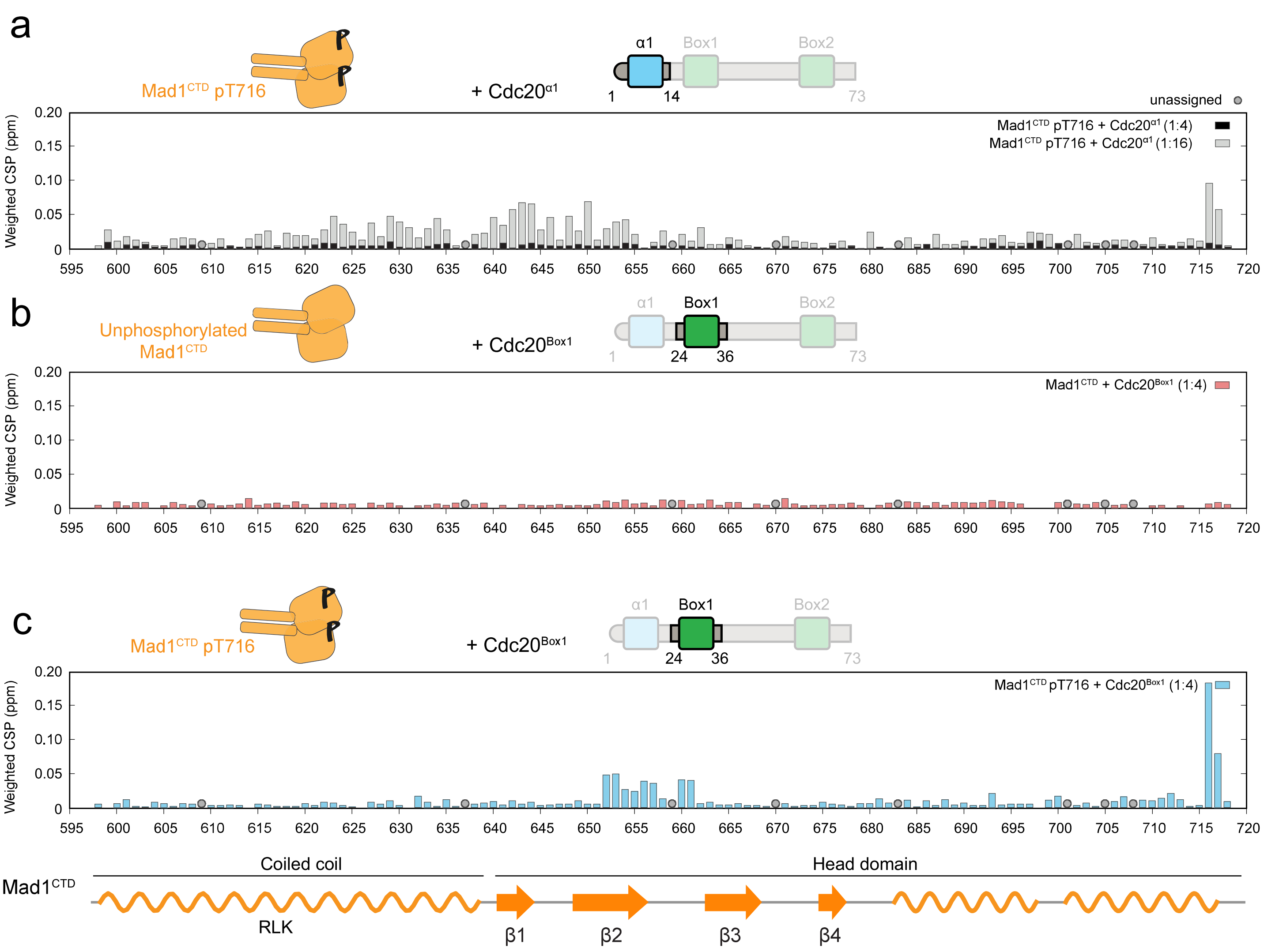

a
Mad1CTD pT716
+ Cdc20α1
α1
Box1
Box2
1
14
73
unassigned
Mad1CTD pT716 + Cdc20α1 (1:4)
Mad1CTD pT716 + Cdc20α1 (1:16)
Weighted CSP (ppm)
0.20
0.15
0.10
0.05
0
595
600
605
610
615
620
625
630
635
640
645
650
655
660
665
670
675
680
685
690
695
700
705
710
715
720
b
Unphosphorylated Mad1CTD
+ Cdc20Box1
24
36
Mad1CTD + Cdc20Box1 (1:4)
c
Mad1CTD pT716
+ Cdc20Box1
Mad1CTD pT716 + Cdc20Box1 (1:4)
Mad1CTD
Coiled coil
Head domain
RLK
β1
β2
β3
β4

Supp Fig 6: Data analysis for titrations of Cdc20$^{\alpha1}$ and Cdc20^{Box1} peptides to ^{15}N-labelled Mad1^{CTD}. Weighted chemical shift perturbations from the ^{1}H, ^{15}N 2D HSQC of phosphorylated or unphosphorylated Mad1^{CTD} were mapped onto the Mad1^{CTD} sequence. Only chemical shift perturbations are shown because no significant change in peak intensity was observed. The schematics above each plot indicate the proteins used in each experiment. Unassigned peaks are denoted as grey circles. Secondary structure of Mad1^{CTD} is shown in the schematics below the plots. **a** Titrating Cdc20$^{\alpha1}$ into Mad1^{CTD} pT716 from Supp Fig 3c. Spectra with two concentrations of titrants were analyzed, 1:4 in black and 1:16 in grey. It should be emphasized that these interactions are weak and CSPs were often only observable at high excess of Cdc20$^{\alpha1}$. **b** Titrating Cdc20^{Box1} into Mad1^{CTD} pT716 from Supp Fig 3d. **c** Titrating Cdc20^{Box1} into unphosphorylated Mad1^{CTD} from Supp Fig 3e. For the Cdc20^{Box1} titrations, spectra with the highest concentration of titrants (1:4) were analysed.

Supp Fig 7

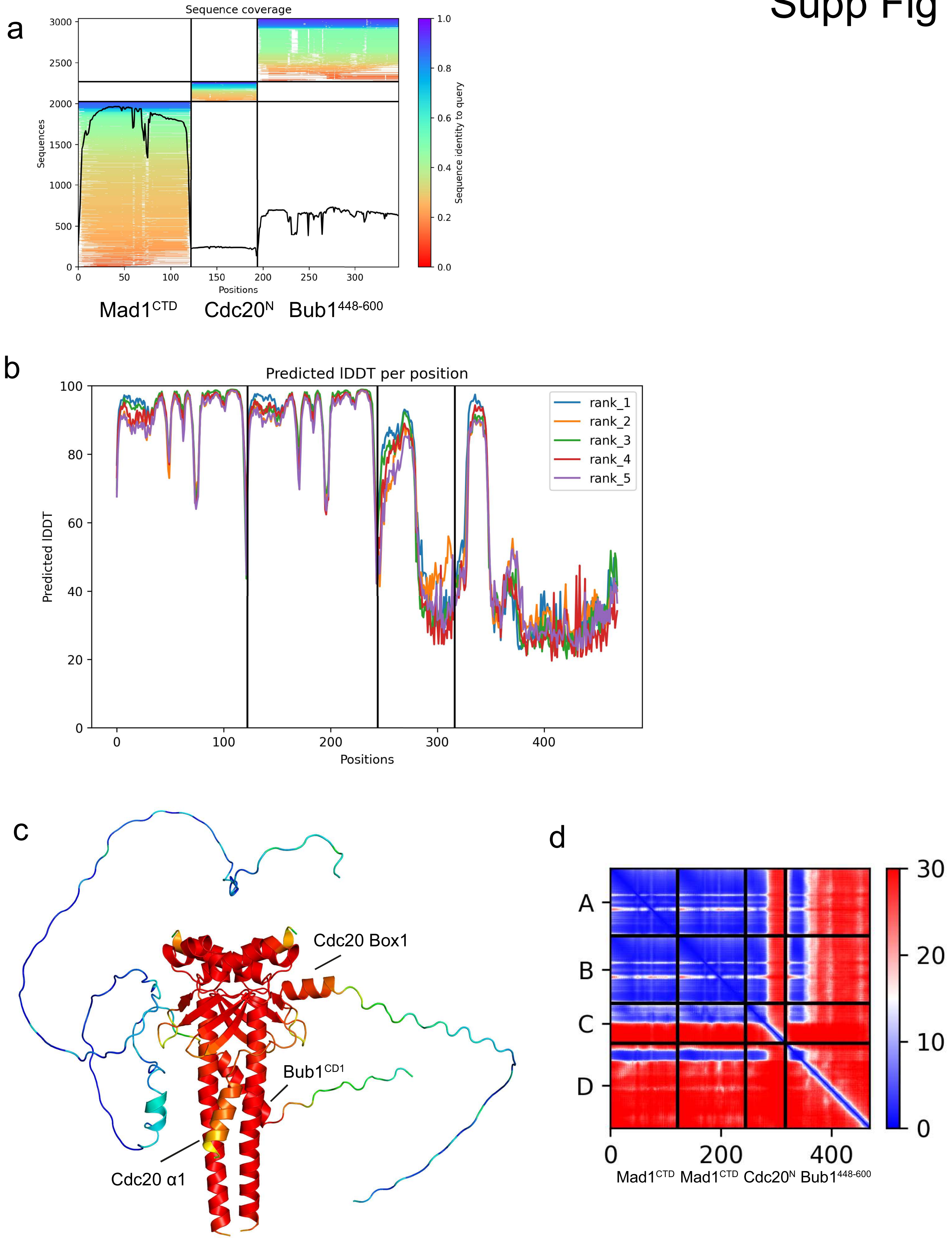

a
Sequence coverage
Sequences
Positions
Sequence identity to query
Mad1CTD Cdc20N Bub1448-600
b
Predicted lDDT per position
Predicted lDDT
Positions
rank_1
rank_2
rank_3
rank_4
rank_5
c
Cdc20 Box1
Bub1CD1
Cdc20 α1
d
A
B
C
D
Mad1CTD Mad1CTD Cdc20N Bub1448-600

Supp Fig 7: Confidence metrics for the Bub1$^{448\text{-}600}$:Mad1^{CTD}:Cdc20^{N} AlphaFold2 prediction. a Coverage plot for the AlphaFold2 prediction multisequence alignment. Position 1-122, 123-196 and 197-350 correspond to residues 597-718 in Mad1, 1-73 in Cdc20 and 448-600 in Bub1, respectively. **b** Predicted local distance difference test (pLDDT) values for the AlphaFold2 model of Bub1$^{448\text{-}600}$:Mad1^{CTD}:Cdc20^{N}. A higher score indicates higher confidence in the prediction. **c** The pLDDT values mapped onto the predicted model of Bub1$^{448\text{-}600}$:Mad1^{CTD}:Cdc20^{N}. Residues are coloured in a scale of high (red) to low (blue) pLDDT values. The Mad1^{CTD} homodimer, Bub1^{CD1}, Cdc20$^{\alpha 1}$ and Cdc20^{Box1} were all predicted with high confidence (pLDDT > 80). A region following Bub1^{CD1} was predicted to be α-helical (cyan) but with relatively low confidence (pLDDT < 60). **d** The predicted alignment error (PAE) heat map for Bub1$^{448\text{-}600}$:Mad1^{CTD}:Cdc20^{N} AlphaFold2 prediction. The PAE heat map shows the predicted error (in angstroms) between all pairs of residues, with blue indicating lower error and red indicating higher error. The PAE plot suggests high confidence for the predicted interactions between Mad1^{CTD}:Cdc20^{N} and Mad1^{CTD}:Bub1^{CD1}.

Supp Fig 8

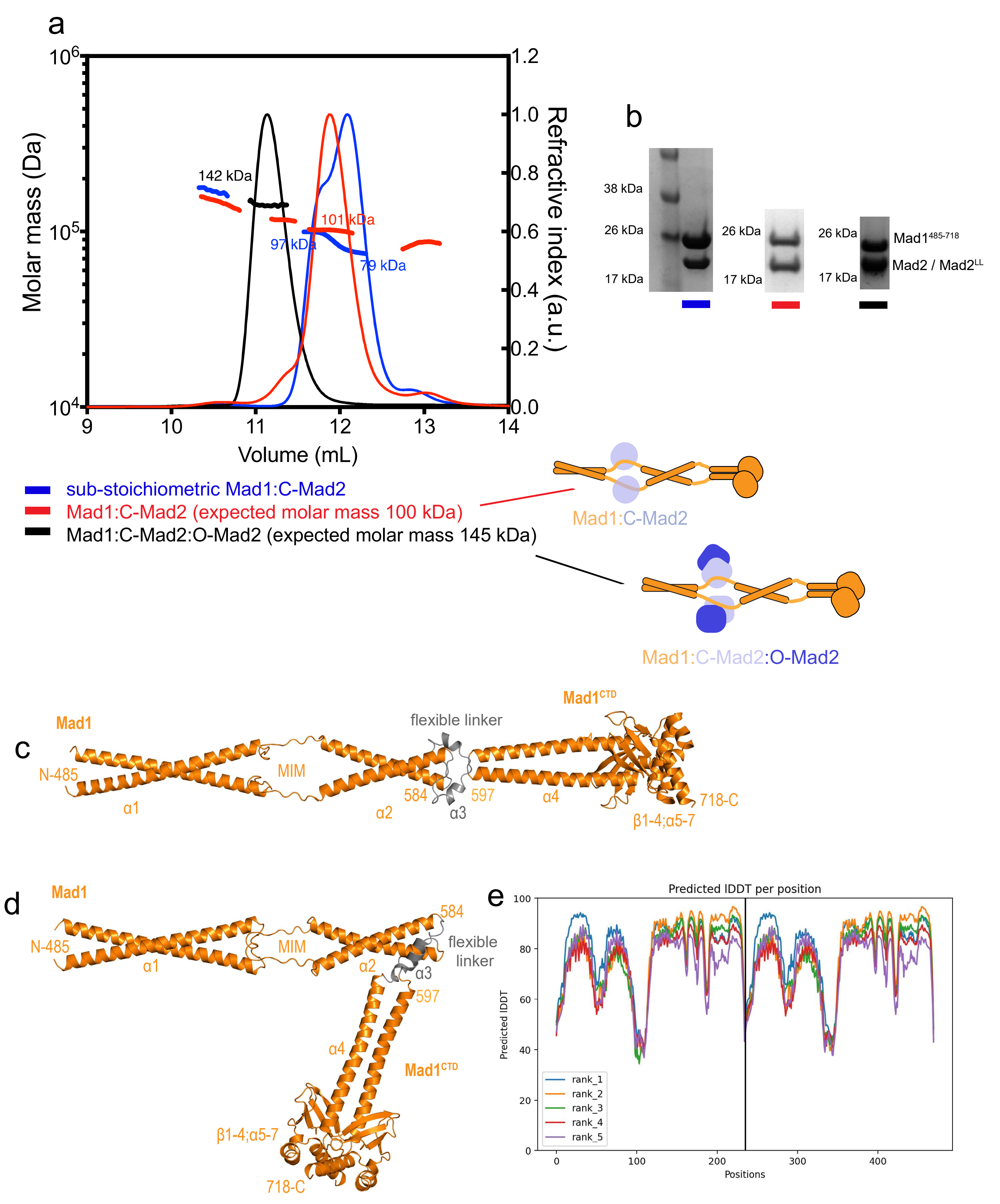

a
Molar mass (Da)
Refractive index (a.u.)
Volume (mL)
142 kDa
101 kDa
97 kDa
79 kDa
sub-stoichiometric Mad1:C-Mad2
Mad1:C-Mad2 (expected molar mass 100 kDa)
Mad1:C-Mad2:O-Mad2 (expected molar mass 145 kDa)
Mad1:C-Mad2
Mad1:C-Mad2:O-Mad2
b
38 kDa
26 kDa
17 kDa
Mad1485-718
Mad2 / Mad2LL
c
Mad1
Mad1CTD
flexible linker
N-485
MIM
α1
α2
584
597
α3
α4
718-C
β1-4;α5-7
d
e
Predicted lDDT per position
Predicted lDDT
Positions
rank_1
rank_2
rank_3
rank_4
rank_5

Supp Fig 8: Formation of homogenous Mad1:Mad2 complexes and AlphaFold2 protein structure prediction for the Mad1 dimer. a SEC-MALS analysis of purified Mad1:Mad2 complexes, where Mad1 only contains residues 485-718. In blue is the Mad1:C-Mad2 complex purified from insect cells which is a sub-stoichiometric mixture of Mad1 dimer (expected 53 kDa), Mad1 dimer with one Mad2 molecule (expected 77 kDa), and Mad1:C-Mad2 tetramer (expected 100 kDa). In red is the homogenous Mad1:C-Mad2 tetramer after incubating the complex with excess Mad2 WT and then re-performing SEC. In black is the homogenous Mad1:C-Mad2:O-Mad2 hexamer after incubating homogenous tetramer with excess O-Mad2 (using the Mad2LL mutant described in the methods section) and re-performing SEC. Three independent measurements with similar results of each sample was completed. **b** The Mad1-Mad2 complexes outlined in (a) analysed by SDS-PAGE. **c,d** AlphaFold2 predicts the $Mad1^{485\text{-}718}$ dimer can exist in both an open-state (**c**) or a folded-state (**d**). Mad1 residues 485-584 and 597-718 are coloured orange, which includes the MIM motif. A novel short helix is predicted in the loop which disrupts the coiled-coil of α2 and α4 and is coloured grey. **e** Predicted local distance difference test (pLDDT) values for the predicted models of Mad1 dimer. A higher score indicates higher confidence in the prediction. Rank 1 model in blue is the highest ranked folded state (d) and the Rank 2 model in orange is the elongated state (c).

Supp Fig 9

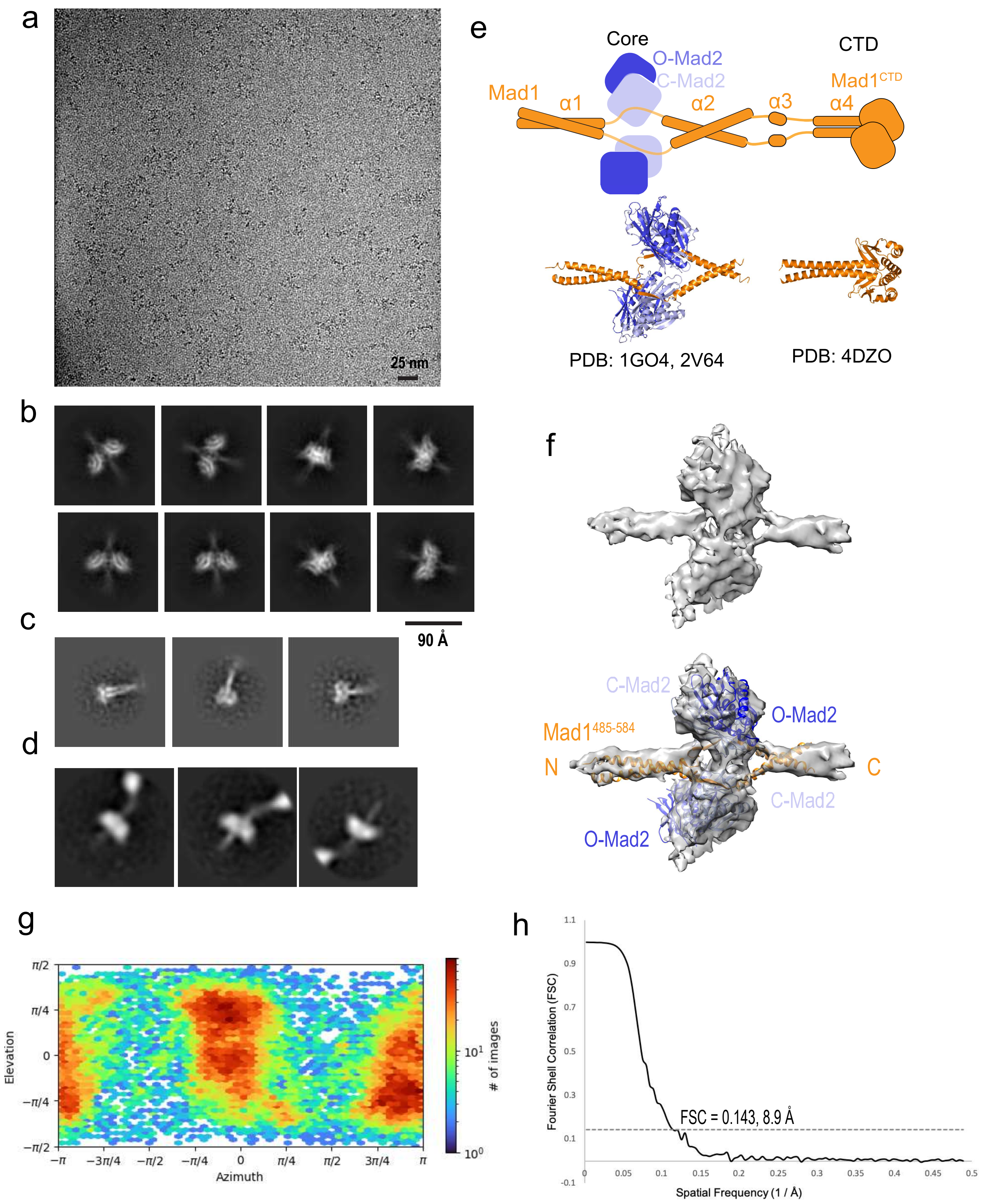

a
25 nm
b
c
90 Å
d
g
Elevation
Azimuth
# of images
e
Core
CTD
O-Mad2
C-Mad2
Mad1
α1
α2
α3
α4
Mad1CTD
PDB: 1GO4, 2V64
PDB: 4DZO
f
C-Mad2
O-Mad2
Mad1485-584
N
C
C-Mad2
O-Mad2
h
Fourier Shell Correlation (FSC)
FSC = 0.143, 8.9 Å
Spatial Frequency (1 / Å)

Supp Figure 9: Cryo-EM of unphosphorylated Mad1$^{485\text{-}718}$:C-Mad2:O-Mad2. a A representative cryo-EM micrograph from 890 total of the BS3 cross-linked Mad1:C-Mad2:O-Mad2 complex collected on a Titan Krios (ThermoFisher Scientific) 300 kV microscope equipped with a K2 detector (Gatan) in counting mode. **b** 2D averages, representative of 35 2D averages, of Mad1:C-Mad2:O-Mad2 capturing the rigid Mad2 core and the N-terminal coiled-coil and C-terminal α-helices. **c** Representative 2D averages of Mad1:C-Mad2:O-Mad2 capturing the C-terminal domain of Mad1 (Mad1^{CTD}). **d** Increasing the box size from 150 Å to 260 Å produces 2D averages which show Mad1^{CTD} flexibly tethered to the core. **e** Model diagram of Mad1:C-Mad2:O-Mad2 and the equivalent crystal structures. **f** A view of the 3D reconstruction of Mad1:C-Mad2:O-Mad2 core at 8.9Å, with the crystal structures (PDBs 1GO4 and 2V64)[23,26] fitted into the cryo-EM reconstruction below. **g** An angular distribution map calculated in cryoSPARC for particle projections. The heat map identifies the number of particles for each viewing angle. This map suggests there is a preferred orientation for the unphosphorylated Mad1:C-Mad2:O-Mad2 complex. **h** A gold-standard fourier shell correlation (FSC) of the Mad1:C-Mad2:O-Mad2 core reconstruction.

Supp Fig 10

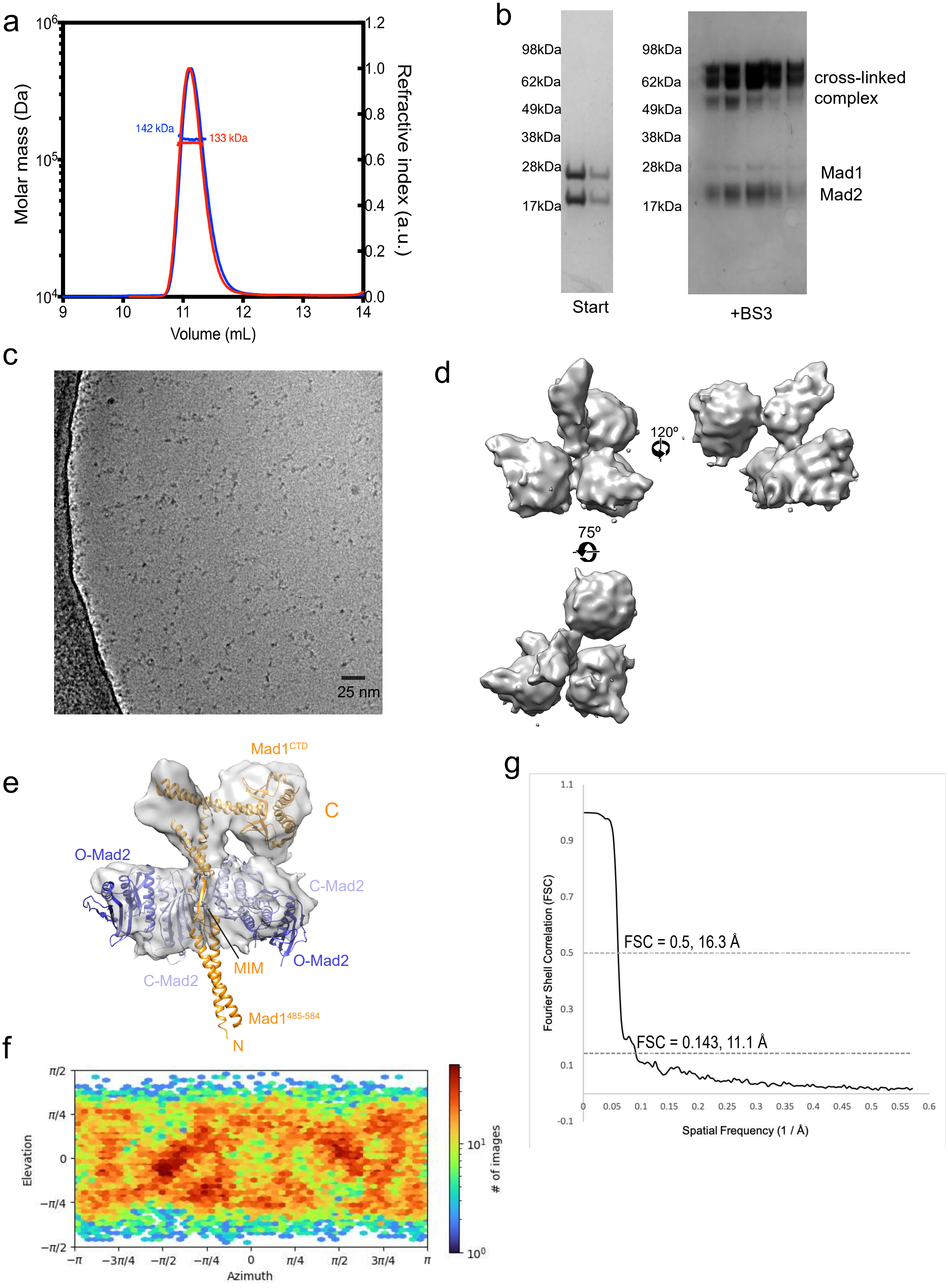
a
Molar mass (Da)
Refractive index (a.u.)
Volume (mL)
142 kDa
133 kDa
b
98kDa
62kDa
49kDa
38kDa
28kDa
17kDa
Start
+BS3
cross-linked complex
Mad1
Mad2
c
25 nm
d
120º
75º
e
Mad1CTD
C
O-Mad2
C-Mad2
MIM
Mad1485-584
N
f
Elevation
Azimuth
# of images
g
Fourier Shell Correlation (FSC)
FSC = 0.5, 16.3 Å
FSC = 0.143, 11.1 Å
Spatial Frequency (1 / Å)

Supp Fig 10: Supplemental cryo-EM data of phosphorylated Mad1$^{485\text{-}718}$:C-Mad2:O-Mad2. a SEC-MALS analysis of pMad1:C-Mad2:O-Mad2 before (blue) and after BS3 (red) cross-linking (expected 145 kDa). Three independent measurements were made with similar results. **b** SDS-page analysis of pMad1:C-Mad2:O-Mad2 before and after cross-linking. **c** A representative cryo-EM micrograph from 3,672 total of the pMad1:C-Mad2:O-Mad2 complex collected on a Titan Krios (ThermoFisher Scientific) 300 kV microscope equipped with a K3 detector and volta-phase plate. **d** Several views of the 3D reconstruction of the pMad1:C-Mad2:O-Mad2 complex. **e** A 3D reconstruction of pMad1:C-Mad2:O-Mad2 in a folded state with the crystal structures manually fitted into the electron density (PDBs 4DZO, 1GO4, 2V64)[23,24,26]. **f** An angular distribution map calculated in cryoSPARC for particle projections. The heat map identifies the number of particles for each viewing angle. No preferred orientation is seen, unlike the unphosphorylated Mad1:C-Mad2:O-Mad2 complex (Supp Fig 9g). **g** FSC of the pMad1:C-Mad2:O-Mad2 core reconstruction.

Supp Fig 11

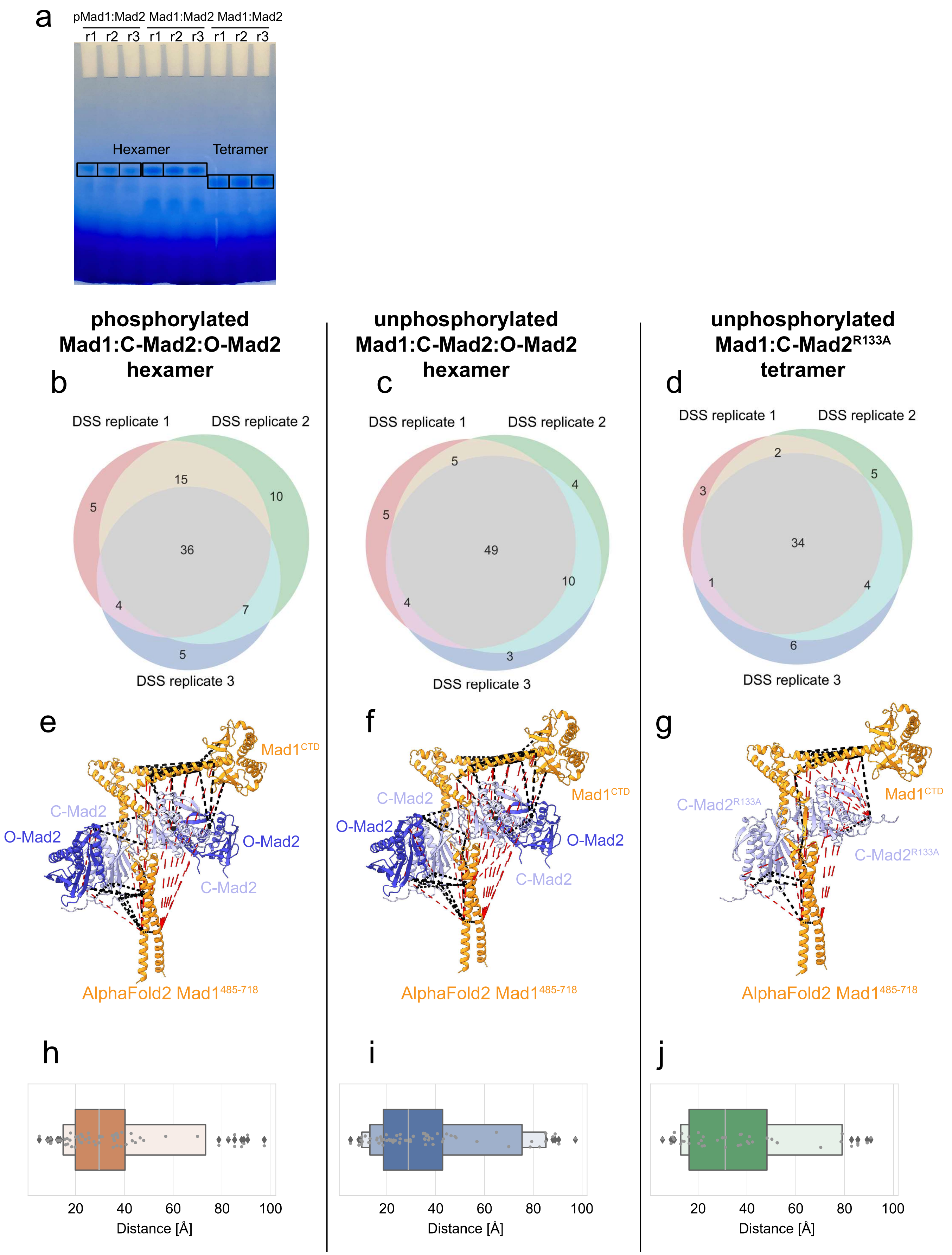

a
pMad1:Mad2
Mad1:Mad2
Mad1:Mad2
r1 r2 r3 r1 r2 r3 r1 r2 r3
Hexamer
Tetramer
phosphorylated Mad1:C-Mad2:O-Mad2 hexamer
unphosphorylated Mad1:C-Mad2:O-Mad2 hexamer
unphosphorylated Mad1:C-Mad2R133A tetramer
b
c
d
DSS replicate 1
DSS replicate 2
DSS replicate 3
15
5
10
36
4
7
5
5
4
5
49
10
4
3
2
3
5
34
1
4
6
e
f
g
Mad1CTD
C-Mad2
O-Mad2
C-Mad2R133A
AlphaFold2 Mad1485-718
h
i
j
20
40
60
80
100
Distance [Å]

Supp Fig 11: Overview of Mad1:Mad2 as analysed by IGX-MS. a Blue Native-PAGE of the three Mad1:Mad2 complexes analysed. Each of the boxed bands were excised, and in-gel cross-linking was performed providing three independent replicates of each sample with similar results. Homogenous Mad1:C-Mad2 tetramer formed using a dimerization deficient R133A Mad2 mutant described in the methods section was used to analyse the tetramer and ensure no super-stoichiometric amount of Mad2 was present. Venn diagrams showing the reproducibility of the IGX-MS experiments, for phosphorylated Mad1:C-Mad2:O-Mad2 (**b**), unphosphorylated Mad1:C-Mad2:O-Mad2 (**c**), and unphosphorylated Mad1:C-Mad2^{R133A} (**d**). Cross-links obtained for each sample are mapped onto the corresponding AlphaFold2 models for phosphorylated Mad1:C-Mad2:O-Mad2 (**e**), unphosphorylated Mad1:C-Mad2:O-Mad2 (**f**), and unphosphorylated Mad1:C-Mad2^{R133A} (**g**). The models were aligned onto Mad1^{MIM} using PDB 1GO4[23] for C-Mad2 and PDB 2V64[26] for O-Mad2. Mapped cross-links with a distance below 40 Å are indicated as black dashed lines and all others in red. Mad1 subunits (residues 485-718) are shown in orange, whereas closed and open Mad2 subunits (C-Mad2/O-Mad2) are coloured in light and dark blue, respectively. For all obtained cross-links, only the shortest option is depicted. **h-j** Boxen plots[81] for phosphorylated Mad1:C-Mad2:O-Mad2 (**h**), unphosphorylated Mad1:C-Mad2:O-Mad2 (**i**), and unphosphorylated Mad1:C-Mad2^{R133A} (**j**). The Boxen plots (explained in the methods section) show the obtained cross-link distances as grey dots, with outliers marked with a dark grey diamond for each sample and are used to improve visualization of the distribution of cross-link distances for Mad1:Mad2 complexes which are highly flexible and thus have more outliers. The cross-links shown are those detected in 2 out of 3 independent IGX-MS experiments. 41 total cross-links were detected in unphosphorylated Mad1:C-Mad2 tetramer, and 68 and 62 cross-links in unphosphorylated and phosphorylated Mad1:C-Mad2:O-Mad2 hexamer, respectively.

Supp Fig 12

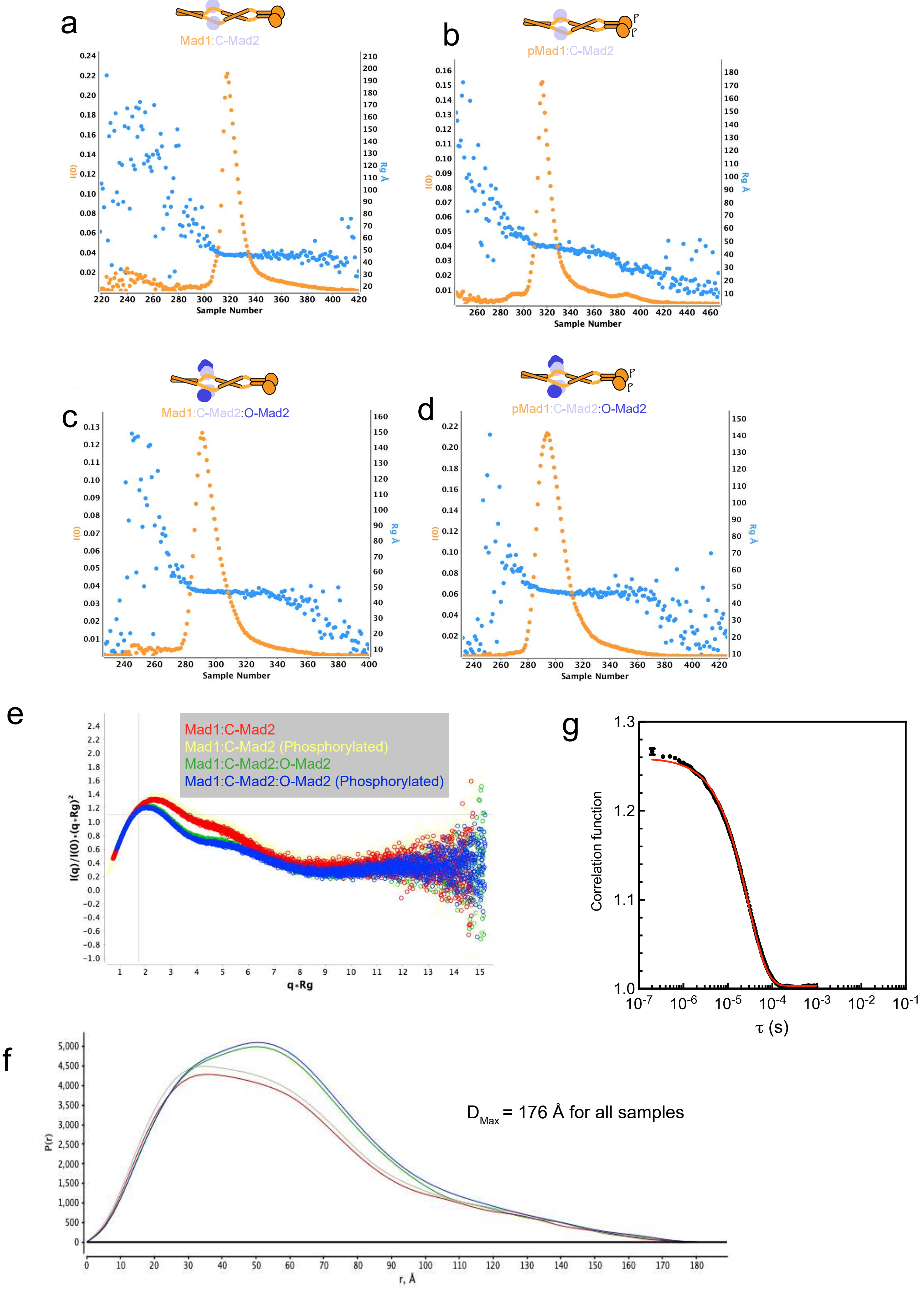
a
Mad1:C-Mad2
I(0)
Rg Å
Sample Number
b
pMad1:C-Mad2
I(0)
Rg Å
Sample Number
c
Mad1:C-Mad2:O-Mad2
I(0)
Rg Å
Sample Number
d
pMad1:C-Mad2:O-Mad2
I(0)
Rg Å
Sample Number
e
Mad1:C-Mad2
Mad1:C-Mad2 (Phosphorylated)
Mad1:C-Mad2:O-Mad2
Mad1:C-Mad2:O-Mad2 (Phosphorylated)
I(q)/I(0)*(q*Rg)²
q*Rg
g
Correlation function
τ (s)
f
P(r)
r, Å
D_{Max} = 176 Å for all samples

Supp Fig 12: SEC-SAXS and QELS analysis of Mad1:Mad2 complexes. a-d The SEC profile with I(0) (intensity) in orange and R_g (radius of gyration) in blue estimated from the Guinier region for each subtracted frame and monitored over the entire elution peak. All Mad1:Mad2 complexes eluted as a single peak and showed a high degree of compositional homogeneity with an average R_g across the peak of 46 Å. Unphosphorylated Mad1:C-Mad2^{R133A} (a), phosphorylated Mad1:C-Mad2^{R133A} (b), unphosphorylated Mad1:C-Mad2:O-Mad2 (c), phosphorylated Mad1:C-Mad2:O-Mad2 (d). **e** A normalized Kratky plot, showing $(q \times R_g)^2 \times I(q)\backslash I(0)$ versus $q \times R_g$ where q is the spatial frequency in 1/Å, I(q) is the scattering intensity at that spatial frequency and R_g is the radius of gyration. Kratky plots can be used as a qualitative assessment of sample flexibility and suggests that the hexameric complex containing O-Mad2 is slightly less flexible than the tetrameric complex but that there is no clear difference between the phosphorylated and unphosphorylated samples. **f** The pairwise distribution function (P(r) function), calculated from the SAXS intensity curve. All pairwise distances (r, Å) between scattering points within the molecule are plotted and extend to an approximate D_{max} of 176 Å for all Mad1:Mad2 complexes. The P(r) was normalized to volume. This suggests that there is no significant difference in conformation between the samples. A fully-extended Mad1:Mad2 structure has an estimated maximum distance of 202 Å, while the folded model has an estimated maximum distance of 151 Å. **g** SEC-MALS dynamic light scattering of pMad1:C-Mad2:O-Mad2. Typical autocorrelation function from the QELS (quasi-elastic light scattering) detector of a single timepoint in the chromatographic peak in Supp Fig 8a. The Rh was determined from the fitted translational diffusion coefficient averaged across the peak (Rh = 53 Å ± 1 = 2.4%).

Supp Fig 13

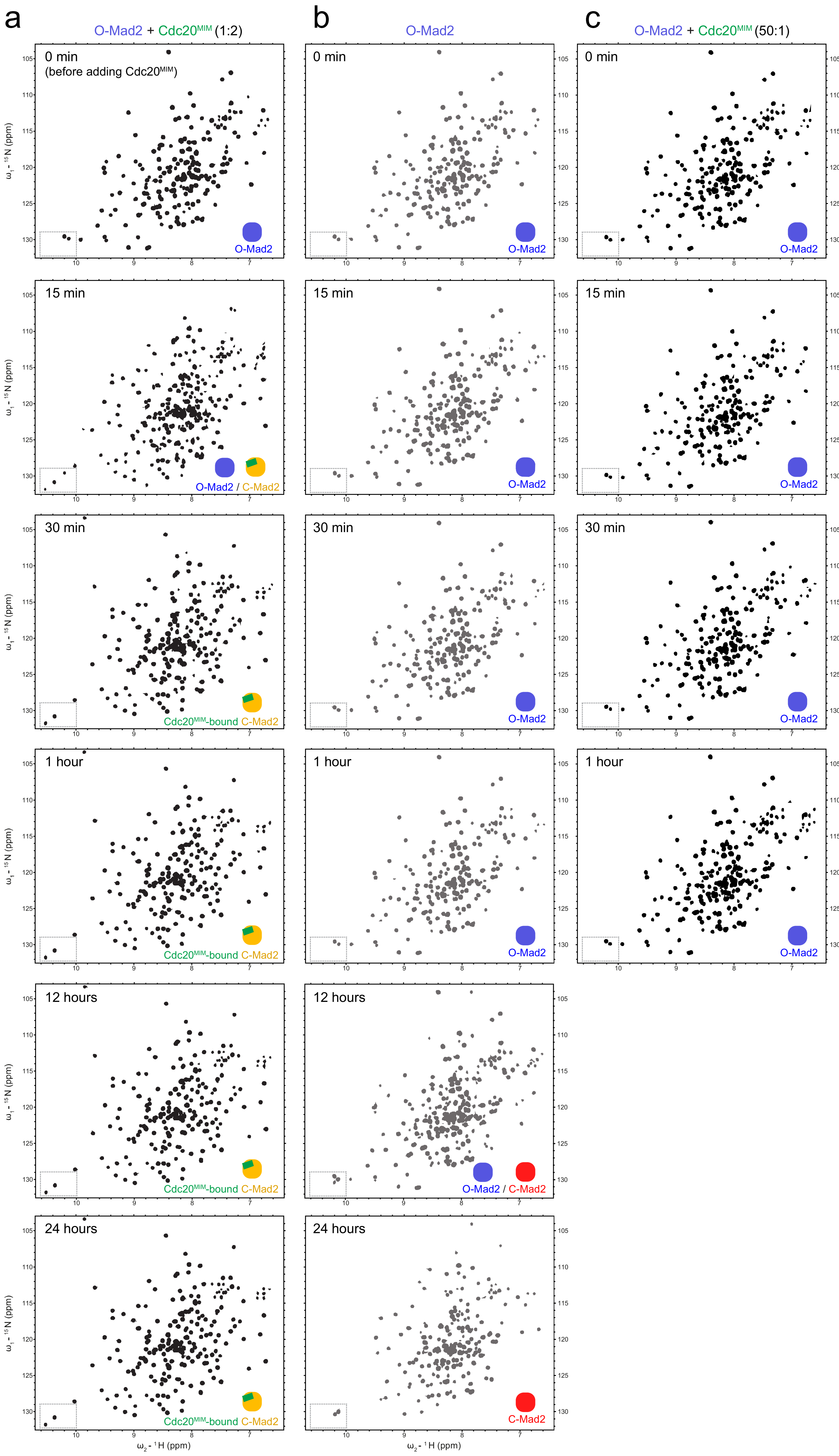
a
O-Mad2 + Cdc20MIM (1:2)
b
O-Mad2
c
O-Mad2 + Cdc20MIM (50:1)
0 min
(before adding Cdc20MIM)
15 min
30 min
1 hour
12 hours
24 hours
O-Mad2
O-Mad2 / C-Mad2
Cdc20MIM-bound C-Mad2
C-Mad2
ω1 - 15N (ppm)
ω2 - 1H (ppm)

Supp Fig 13: The MIM of Cdc20 induces Mad2 conversion. ^{1}H, ^{15}N 2D HSQC of ^{15}N-labelled O-Mad2 during open-to-closed conversion of Mad2. **a,b** Conversion of 100 µM ^{15}N-labelled O-Mad2 R133A in the presence (a) or absence (b) of 200 µM $Cdc20^{MIM}$ peptide at 25°C. As shown in Figure 7a, the side chains of Trp167 and Trp75 are diagnostic of Mad2 conformation, and their resonances are boxed in the spectra. The schematics show the conformation of Mad2 as indicated by the tryptophan side chain resonances. **c** Substoichiometric concentration of $Cdc20^{MIM}$ was used in (c) where 2 µM of $Cdc20^{MIM}$ was added to 100 µM ^{15}N-labelled O-Mad2 R133A. The majority of Mad2 remains in an open state, in marked contrast to the spontaneous conversion observed when $Cdc20^{MIM}$ was added in a 2-fold excess (a). This suggests that $Cdc20^{MIM}$ cannot be reused in the reaction and is therefore not a catalyst for Mad2 conversion. Instead $Cdc20^{MIM}$ functions to induce conversion and stabilizes $Cdc20^{MIM}$-bound C-Mad2.

Supp Fig 14

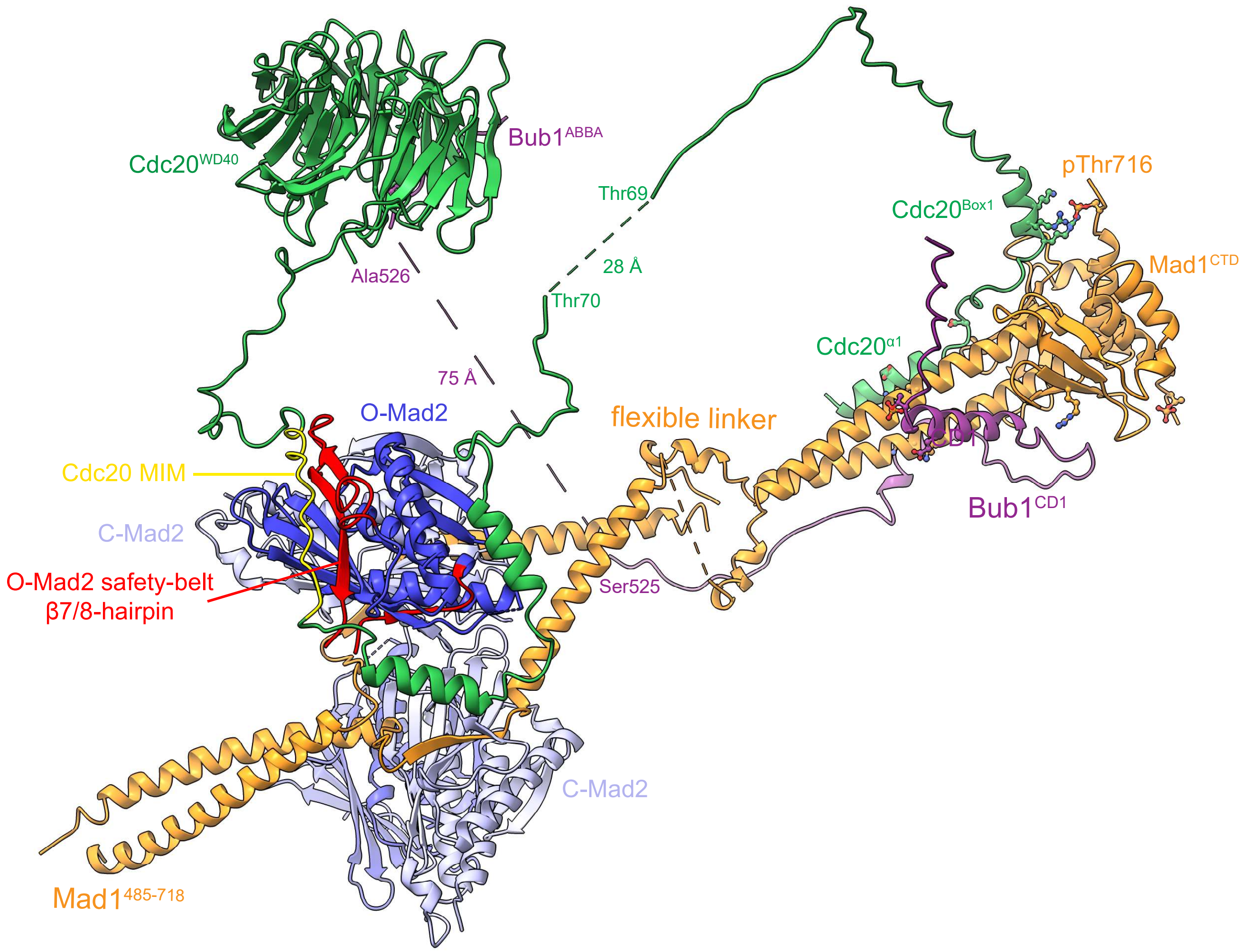
Cdc20^{WD40}
Bub1^{ABBA}
Thr69
28 Å
Thr70
Ala526
75 Å
pThr716
Cdc20^{Box1}
Mad1^{CTD}
Cdc20$^{\alpha1}$
O-Mad2
flexible linker
Cdc20 MIM
C-Mad2
Bub1^{CD1}
O-Mad2 safety-belt
β7/8-hairpin
Ser525
C-Mad2
Mad1$^{485-718}$

Supp Fig 14. Model of pBub1:Cdc20:pMad1:C-Mad2:O-Mad2 when Mad1 is in the elongated state. As compared to when Mad1 is in the folded state (Fig 7c). The model was generated using PDBs 1GO4, 2V64, 6TLJ, 7B1F[21,23,26,90] and the AlphaFold2 models of Bub1$^{448-534}$:Mad1^{CTD}:Cdc20 and the folded model of Mad1$^{485-718}$, and displayed using ChimeraX. Residues for the Mad1 pThr716 and Cdc20^{Box1} Arg27/Arg30/Lys31, Cdc20$^{\alpha1}$ Asp9 and Mad1 Lys619, Mad1 Arg617 and Bub1 pThr461 interactions are depicted as sticks.

Supp Fig 15

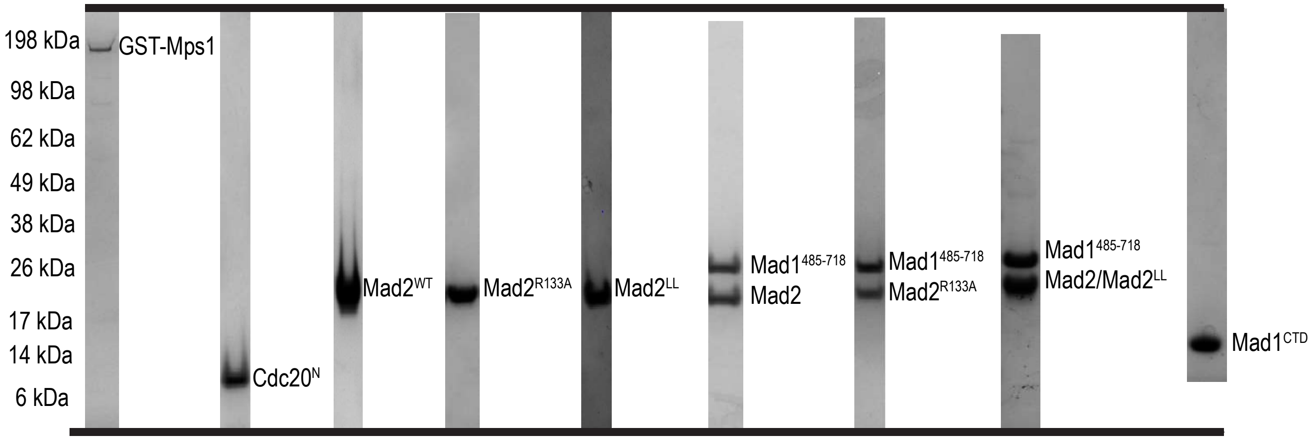

198 kDa
98 kDa
62 kDa
49 kDa
38 kDa
26 kDa
17 kDa
14 kDa
6 kDa
GST-Mps1
Cdc20^{N}
Mad2^{WT}
Mad2^{R133A}
Mad2^{LL}
Mad1$^{485-718}$
Mad2
Mad1$^{485-718}$
Mad2^{R133A}
Mad1$^{485-718}$
Mad2/Mad2^{LL}
Mad1^{CTD}

Supp Fig 15. Gallery of proteins purified in this study. All proteins were analysed by SDS-PAGE post-purification using a 4-12% Bis-Tris Glycine gel with the SeeBlue™ Plus2 Protein Ladder (ThermoFisher Scientific). Mad2LL is a mutant of Mad2 kinetically locked in the open state (O-Mad2)[26]. Mad2 R133A is a dimerization deficient mutant of Mad2 which can still bind Mad1[23].

Supp Table 1

Supp Table 1. Phosphorylation sites within Mad1^{CTD} and Mad1$^{485-718}$ when phosphorylated by full-length GST-Mps1 or the Mps1 kinase domain *in vitro*. The data obtained were compared with two prior studies (1)[ref 14] and (2)[ref 34]. For each phosphosite in this table the number of peptide spectral matches (PSMs) of the phosphorylated and corresponding unphosphorylated peptide is provided, as a semi-quantitative indication of the site occupancy. Residues which were found to be phosphorylated are shaded orange with the Thr716 site coloured in a darker shade of orange, being the only site with much more PSMs for the phosphorylated *versus* the non-phosphorylated peptide. No phosphorylated peptides were found in any of the samples prior to the *in vitro* kinase assay treatment.

Phosphorylation sites<br>Kinase	Mad1$^{485-718}$<br>GST-Mps1	Mad1$^{485-718}$<br>Mps1ΔKD	Mad1^{CTD}<br>GST-Mps1	Mad1^{CTD}<br>Mps1ΔKD	Comparison with published studies
S485		1/389			1
S486		2/290			
S490		4/170			1
S494					1
T500	1/5	2/7			1
Y535					1
S538	17/121	8/46			1
T540	10/76	30/0			1
S546		2/104			1
T550	7/345	11/431			1
S551	5/11	4/17			1
S562					
S594		3/109			
S597		2/79			
S583		12/201			1
S598					1, 2
S610					2
T624					2
T644	1/200	1/212	2/300	1/103	1
T645		1/190			
S699	2/561	1/141			
S705		2/461			
T716	121/0	33/1	45/2	71/3	1, 2

Supp Table 2

Supp Table 2. Cryo-EM data-collection and 3D modelling statistics.

	**Mad1$^{485\text{-}718}$:C-Mad2:O-Mad2**	**pMad1$^{485\text{-}718}$:C-Mad2:O-Mad2**
Microscope	FEI Titan Krios G2	FEI Titan Krios G2
Voltage (kV)	300	300
Detector	Gatan K2	Gatan K3
Nominal magnification	105,000x	105,000x
Pixel size (Å)	1.1	0.86
Volta phase plate	No	Yes
Electron dose per frame (e^-/A^2)	1.03	1.05
Defocus range	-1.4 to -2.4	-0.5 to -1.0
Number of micrographs	890	3,672
Total number of picked particles	37,671	210,527
Number of particles used in reconstruction	13,805	33,104
Final resolution (Å)	8.9 at 0.143 FSC	11.1 at 0.143 FSC<br>16.3 at 0.5 FSC
