# Supplementary material for: Juxtaposition of Bub1 and Cdc20 on phosphorylated Mad1 during catalytic mitotic checkpoint complex assembly
Source: Nat Commun. 2022 Oct 26;13:6381. doi: 10.1038/s41467-022-34058-2 (PMC9605988; doi:10.1038/s41467-022-34058-2)
Supplement: Supplementary file 3 — Description of Additional Supplementary Files [file 41467_2022_34058_MOESM3_ESM.pdf]

Description of Additional Supplementary Files

File name: Supplementary Data 1

Description: PDB coordinates for the model of pBub1:Cdc20:pMad1:C-Mad2:O-Mad2 complex (MCC-assembly scaffold) posed for MCC formation. The model was generated using PDBs 1GO4, 2V64, 6TLJ, 7B1F[21,23,26,90] and the AlphaFold2 models of Bub1$^{448-534}$:Mad1^{CTD}:Cdc20 and the folded model of Mad1$^{485-718}$.

File name: Supplementary Movie 1

Description: Rocking movie of the MCCassembly scaffold model shown in main text Figure 7C.
