# Supplementary material for: Juxtaposition of Bub1 and Cdc20 on phosphorylated Mad1 during catalytic mitotic checkpoint complex assembly
Source: Nat Commun. 2022 Oct 26;13:6381. doi: 10.1038/s41467-022-34058-2 (PMC9605988; doi:10.1038/s41467-022-34058-2)
Supplement: Supplementary file 6 — Reporting Summary [file 41467_2022_34058_MOESM6_ESM.pdf]

nature portfolio

Corresponding author(s): Elyse Fischer and David Barford

Last updated by author(s): September 30th, 2022

Reporting Summary

Nature Portfolio wishes to improve the reproducibility of the work that we publish. This form provides structure for consistency and transparency in reporting. For further information on Nature Portfolio policies, see our Editorial Policies and the Editorial Policy Checklist.

Statistics

For all statistical analyses, confirm that the following items are present in the figure legend, table legend, main text, or Methods section.

n/a	Confirmed	
☐	☒	The exact sample size (n) for each experimental group/condition, given as a discrete number and unit of measurement
☒	☐	A statement on whether measurements were taken from distinct samples or whether the same sample was measured repeatedly
☒	☐	The statistical test(s) used AND whether they are one- or two-sided<br>*Only common tests should be described solely by name; describe more complex techniques in the Methods section.*
☒	☐	A description of all covariates tested
☒	☐	A description of any assumptions or corrections, such as tests of normality and adjustment for multiple comparisons
☐	☒	A full description of the statistical parameters including central tendency (e.g. means) or other basic estimates (e.g. regression coefficient) AND variation (e.g. standard deviation) or associated estimates of uncertainty (e.g. confidence intervals)
☒	☐	For null hypothesis testing, the test statistic (e.g. F, t, r) with confidence intervals, effect sizes, degrees of freedom and P value noted<br>*Give P values as exact values whenever suitable.*
☒	☐	For Bayesian analysis, information on the choice of priors and Markov chain Monte Carlo settings
☒	☐	For hierarchical and complex designs, identification of the appropriate level for tests and full reporting of outcomes
☒	☐	Estimates of effect sizes (e.g. Cohen's d, Pearson's r), indicating how they were calculated

Our web collection on statistics for biologists contains articles on many of the points above.

Software and code

Policy information about availability of computer code

Data collection	EPU from Thermo Fisher Scientific was used for automated cryo-EM data collection. SEC-MALS data were collected with an Agilent 1200 series LC system with an online Dawn Helios ii system (Wyatt) equipped with a QELS+ module (Wyatt) and an Optilab rEX differential refractive index detector (Wyatt). NMR data were collected on 700 MHz Avance II+ , 800 MHz Avance III and 950 MHz Avance III spectrometer (Bruker). For MS analysis of cross-linked peptides, the samples were re-suspended in 2 % formic acid and analysed using an UltiMate™ 3000 RSLCnano System (ThermoFisher Scientific) coupled on-line to an Orbitrap Exploris 480 (ThermoFischer Scientific).
Data analysis	Cryo-EM data were analysed using the software MotionCor2 v1.1.0, GCTF v1.06, WARP v1.09, RELION v3.0, and CryoSPARC v2.15.0, ChimeraX v1.2.5, Coot v0.8.9.2. Data visualization for SEC-MALS and FP was done in Graphpad Prism v9.4.1. SEC-MALS data were analysed with ASTRA v7.3.0.11 (Wyatt). NMR data were analysed using Topspin v4.1.1 (Bruker) and NMRFAM-Sparky v1.47. Datasets collected with non-uniform sampling were processed with MddNMR v2.7. Raw files obtained for respective IGX-MS experiments were subsequently analysed with the plink 2 software v1.0. Excel 2019 was used. Scaffold v4 was used for identifications made by the Mascot Search Engine from Matrix Software. For Sedimentation Equilibrium SEDPHAT v15.2b was used and data were plotted with GUSSI v1.4.2. When later FP analyses were done, GraphPad Prism v8.3.1 was used. Structure visualization used Chimera X v1.4 and PyMOL v2.5.2. SAXS analyses and visualizations were completed in Scatter IV.

For manuscripts utilizing custom algorithms or software that are central to the research but not yet described in published literature, software must be made available to editors and reviewers. We strongly encourage code deposition in a community repository (e.g. GitHub). See the Nature Portfolio guidelines for submitting code & software for further information.

Data

Policy information about availability of data

All manuscripts must include a data availability statement. This statement should provide the following information, where applicable:

- Accession codes, unique identifiers, or web links for publicly available datasets
- A description of any restrictions on data availability
- For clinical datasets or third party data, please ensure that the statement adheres to our policy

The NMR assignments of Cdc20N were deposited to the BMRB database (http://www.bmrb.wisc.edu/) with the accession number 51304. The in-gel cross-linking mass spectrometry (IGX-MS) data have been deposited to the ProteomeXchange Consortium via the PRIDE (https://www.ebi.ac.uk/pride/) partner repository with the dataset identifier PXD031872. PDBs used in this study: 4DZO, 1GO4, 2V64, 7B1F (https://www.ebi.ac.uk/pdbe). Oligonucleotide primers and the protein sequences used to produce the protein constructs in this study are available from the corresponding authors upon request. Source data are provided with this paper.

Field-specific reporting

Please select the one below that is the best fit for your research. If you are not sure, read the appropriate sections before making your selection.

☒ Life sciences ☐ Behavioural & social sciences ☐ Ecological, evolutionary & environmental sciences

For a reference copy of the document with all sections, see nature.com/documents/nr-reporting-summary-flat.pdf

Life sciences study design

All studies must disclose on these points even when the disclosure is negative.

Sample size	No sample-size calculation was performed. For cryo-EM, the actual sample size was increased until no further improvement in resolution was obtained in the cryo-EM maps. For other experiments, we completed technical replicates or independent experiments and the values given are the means.
Data exclusions	Cryo-EM particle images that fall into 2D averages or 3D classes with poor features were excluded from final reconstruction.
Replication	For AUC-SE experiments the error on the complex mass is from 4 independent replicates. For SEC-MALS the quoted mass is the weighted average across the peak. For fluorescent anisotropy the Kd values are derived from at least 3 separate experiments, with each experiment containing three technical replicates. For IGX-MS 3 technical replicates were performed. For SEC-SAXS, three independent experiments were completed with complex concentration at 9 mg/ml, 6 mg/ml, 3 mg/ml, and then because all results were comparable, the experiment with the strongest signal was included in the manuscript. Replicates which failed due to errors in the baseline or poor signal, etc, were excluded otherwise all replicates were included and 'successful'. All error values are standard errors of the mean value.
Randomization	For cryo-EM, randomization was internally used by data processing software in initial particle assignment for 2D classifications and generation of half maps for resolution estimation. Randomization is not relevant for the IGX-MS-crosslinking, fluorescent anisotropy, SEC-MALS, AUC-SE and NMR experiments in this study.
Blinding	Blinding was not relevant to this study. Blinding is not typically performed for the experiments reported in this paper.

Reporting for specific materials, systems and methods

We require information from authors about some types of materials, experimental systems and methods used in many studies. Here, indicate whether each material, system or method listed is relevant to your study. If you are not sure if a list item applies to your research, read the appropriate section before selecting a response.

Materials & experimental systems

n/a	Involved in the study
☒	☐ Antibodies
☒	☐ Eukaryotic cell lines
☒	☐ Palaeontology and archaeology
☒	☐ Animals and other organisms
☒	☐ Human research participants
☒	☐ Clinical data
☒	☐ Dual use research of concern

Methods

n/a	Involved in the study
☒	☐ ChIP-seq
☒	☐ Flow cytometry
☒	☐ MRI-based neuroimaging
